# Plasminogen mutation–associated thrombotic microangiopathy and role of anticoagulation: a single institution case series

**DOI:** 10.1016/j.rpth.2025.103012

**Published:** 2025-08-13

**Authors:** Shreya Agarwal, Nicola Pozzi, Senthil Sukumar, Camila Masias, Anuja Java, Spero Cataland

**Affiliations:** 1 Division of Hematology, Department of Pediatrics, University of California, San Francisco, California, USA; 2 Department of Biochemistry and Molecular Biology, St. Louis University School of Medicine, St. Louis, Missouri, USA; 3 Division of Hematology/Oncology, Department of Internal Medicine, Baylor College of Medicine, Houston, Texas, USA; 4 Miami Cancer Institute, Baptist Health South Florida, Miami, Florida, USA; 5 Division of Nephrology, Department of Internal Medicine, Washington University, St. Louis, Missouri, USA; 6 Division of Hematology, Department of Internal Medicine, Ohio State University, Columbus, Ohio, USA

**Keywords:** anticoagulation, complement blockade, eculizumab, plasminogen mutation, thrombotic microangiopathy

## Abstract

**Background:**

Knowledge gaps exist regarding the role of coagulation pathway mutations such as those in the plasminogen (*PLG*) gene in the pathogenesis of thrombotic microangiopathy (TMA) and treatment outcomes.

**Objectives:**

This study aims to describe the unique phenotypic features of patients with *PLG* mutations and perform structural mapping of the variants to enhance variant interpretation.

**Methods:**

This was a single-center retrospective study of patients with TMA in whom genetic testing was performed between 2011 and 2023. Data were collected regarding demographics, clinical features at their first presentation, renal outcomes, genetic mutations, and recurrence for those who were found to have a *PLG* mutation. Structural mapping of the *PLG* variants was performed using X-ray structural data.

**Results:**

Over the 12-year study period, we identified 6 individuals in our TMA cohort with *PLG* mutations. Median age at the time of first TMA event was 45.5 years (range: 5-57 years). Nearly all were female (*n* = 5, 83%). Half of the cohort (*n* = 3, 50%) had recurrent TMA, with 1 having recurrent episodes while on long-term complement blockade therapy. Three patients are now on long-term anticoagulation with no further TMA recurrences observed. Structural mapping of the variants revealed that the mutations could be categorized into 3 groups. Among these, group 2 variants (residues K38 in the PAN-apple domain and residue R523 in kringle-5) had a more severe phenotype with severe thrombocytopenia at presentation and a recurrent TMA course.

**Conclusion:**

Patients with *PLG* mutation–associated TMAs appear to have a poor response to complement blockade therapy, suggesting that pathways in addition to or independent of complement dysregulation may be involved in some patients. Future studies are warranted to explore the role of anticoagulation in preventing recurrence in patients with *PLG* mutations.

## Essentials

• Very little is known regarding plasminogen (*PLG*) mutations in thrombotic microangiopathy (TMA).
• We describe 6 patients with TMA with mutations in the *PLG* gene.
• These individuals had recurrent episodes of TMA and did not respond to complement blockade therapy.
• Anticoagulation might be a potential treatment option for this subgroup.

## 1 Introduction

Atypical hemolytic uremic syndrome (aHUS), also known as complement-mediated thrombotic microangiopathy (CM-TMA), is characterized by dysregulation of the alternative complement pathway. It often presents with microangiopathic hemolytic anemia, thrombocytopenia, and multiorgan dysfunction, particularly acute kidney injury (AKI) [1]. At the cellular level, endothelial injury is the hallmark finding [2]. With increasing recognition of the role of complement pathway in TMA pathogenesis [3], the approach to TMA has been transformed over the past decade. The focus is now on early recognition of complement dysregulation and intervention with complement blockade therapy [[4], [5], [6]].

Genetic sequencing in TMA-affected patients has helped to identify genetic variants in complement genes in ∼50% to 60% of individuals [7,8]. While most of the mutations have been shown to occur in the complement pathway, genes in the coagulation pathway, such as *THBD* [9] and *PLG* [10], have also been implicated in the pathogenesis of TMA. Thrombomodulin is encoded by *THBD* and may regulate both the complement and coagulation pathways and has been suggested to play a role in aHUS pathogenesis [9]. Plasminogen, encoded by *PLG*, is a protein that plays an essential role in the fibrinolytic system. While more is known about dysregulation of complement pathway genes, there exist significant knowledge gaps regarding the role of *PLG* mutations in TMA pathogenesis and the treatment outcomes with complement blockade therapy.

Plasminogen is a serine protease (SP) that circulates in the blood plasma as an inactive protein and is converted to its activated form, plasmin, by plasminogen activators such as tissue plasminogen activator. A central role of plasmin is to break down fibrin in blood clots, resulting in dissolution of clots. At the molecular level, circulating plasminogen comprises 7 domains, including 1 PAN-apple (PAN) domain, 5 kringle domains, and 1 SP domain, which come together to adopt a closed/circular conformation [11]. Intramolecular interactions between domains stabilize the closed form, and the kringle domains facilitate recognition of fibrin and cell surface receptors. These interactions can trigger plasminogen to adopt an open conformation, resulting in efficient activation of plasmin by plasminogen activators. Therefore, any mutation in *PLG* that directly or indirectly affects the balance between closed and open forms may change the plasminogen interactome and its functions, potentially increasing the risk of thrombosis [12], including microvascular thrombosis as seen in TMA.

In 2014, Bu et al. [10] conducted a retrospective study to identify complement and coagulation pathway mutations in patients with aHUS using genetic sequencing. They identified 4 variants in *PLG*, 1 novel and 3 previously reported to be associated with a plasminogen deficiency. However, the phenotypic correlation and response to TMA therapy of these variants remains unknown. Here, we report 5 cases of *PLG* mutation–associated TMA and describe our institutional experience and management strategies. Additionally, we characterize the *PLG* variants by performing structural mapping to better understand and interpret the functional consequence of these mutations.

## 2 Methods

### 2.1 Patient cohort and study definitions

This single-center study was conducted at Ohio State University (OSU). All patients enrolled in the OSU TMA registry from January 1, 2011, to December 31, 2023, who had genetic analysis and were found to have a *PLG* mutation were eligible for inclusion in this retrospective study. Patients with confirmed immune thrombotic thrombocytopenic purpura (TTP) were excluded from the study cohort. The OSU TMA registry is approved by the institutional review board.

TMA was diagnosed based on thrombocytopenia (platelet count < 150 × 10^9^/L), microangiopathic hemolytic anemia (hemoglobin <10 g/L, lactate dehydrogenase >500 U/L [normal <190 U/L], and/or presence of schistocytes on peripheral smear), AKI, and non–severely deficient (>10%) ADAMTS-13 level. Thrombocytopenia was categorized as mild (100 to <150 × 10^9^/L), moderate (50 to <100 × 10^9^/L), or severe (<50 × 10^9^/L). Genetic studies were performed using a commercially available targeted next-generation sequencing panel for TMA-associated genes at the University of Iowa Molecular Otolaryngology & Renal Research Laboratories (https://morl.lab.uiowa.edu/). All the genes in this panel are listed in Supplementary Table 1. Patients were followed until death or the date of last clinical contact. Nearly all patients were followed every 6 months in the outpatient clinic with a median follow-up time of 23 months after their first TMA episode.

Data was extracted from the electronic medical records regarding patient demographics, details of initial TMA presentation, TMA gene panel testing results, degree of renal injury, management, and treatment outcomes (renal recovery, TMA relapse, and mortality).

### 2.2 Structural mapping of the *PLG* variants

*PLG* variants related to TMA, either reported in the literature or found in our cohort, were mapped on the closed form of type II Glu-plasminogen, which was experimentally determined at a 2.5 Å resolution by X-ray crystallography in 2012 by Law et al. [11] (PDB ID 4dur, chain B, aa. 20-810). Although 2 additional structures exist (4A5T and 4DUU), this is the highest-resolution structure. Moreover, type II Glu-plasminogen, a glycoform that contains a single *O*-linked glycosylation moiety on Thr346, is the most abundant, comprising ∼60% of the circulating PLG material. Figures were made with PyMOL (Schrödinger, Inc).

### 2.3 Statistical analysis

Descriptive statistics were used to summarize the data.

## 3 Results

### 3.1 Patient demographics and clinical presentation

**Figure 1:**
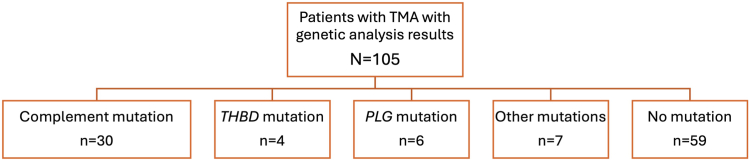
CONSORT diagram for all the patients with TMA seen at Ohio State University with their genetic results. TMA, thrombotic microangiopathy.

A total of 105 individuals with TMA with genetic results were identified (Figure 1). Of these, 6 individuals carried a *PLG* mutation (Table). One individual with a *PLG* mutation also had an *MCP* mutation. Median age at the time of first TMA event was 45.5 years (range: 5-57 years). Five of the 6 patients (83%) were female (*n* = 5, 83%) and none of them developed postpartum TMA, despite multiple pregnancies.

**Table:** Details regarding patients with *PLG* mutation.

Patient no.	Age at first TMA (y)	Gender	*PLG* mutation	Zygosity	Other gene mutation	Plt count (10^9^/L)	Cr (mg/dL)	LDH (U/L)	ESKD at presentation	ESKD at last visit	Relapse	Anticoagulation
1	32	Female	c.112A>G, p.Lys38Glu	heterozygous	MCP/CD46 polymorphism	10	4.28	762	Yes	No	Yes (5); 2 while on eculizumab	Yes (rivaroxaban)
2	55	Female	NM_000301:3c.1567C>T, p.Arg523Trp	heterozygous	–	46	10.4	532	Yes	No	Yes (4)	Yes (apixaban, started after the last recurrence)
3	43	Female	NM_000301.3:c.782G>A, p.Arg261His	heterozygous	del(CFHR3-CFHR1) and del(CFHR1-CFHR4)	145	4.53	592	Yes	Yes	No	No
4	56	Female	NM_000301.3:c.266G>A, p. Arg89Lys	heterozygous	–	56	2.89	503	No	No	No	No
5	36	Female	NM_000301:c.341C>T, p.Thr114Met	heterozygous	–	166	3.66	313	No	No	No	Yes (warfarin)
6	5	Male	NM_000301:c.1114C>G, p.Pro372Ala	heterozygous	CD46 (NM 002389:c.175C>T, p.Arg59Stop)	20	3	380	No	No	Yes (6)	No

Cr, creatinine; ESKD, end stage kidney disease; LDH, lactate dehydrogenase; Plt, platelet.

Fifty percent of the patients had recurrent TMA, with one of them having recurrent TMA episodes while on long-term complement blockade therapy. Three patients are now on long-term anticoagulation therapy with either direct oral anticoagulants (*n* = 2) or warfarin (*n* = 1). Below, we describe in detail each of the 6 patients with *PLG* variants. Residues are numbered according to the gene (P00747_PLMN_HUMAN, aa. 1-810), which includes the signal peptide (aa. 1-19) followed by the mature circulating form of *PLG* (aa. 20-810). Additional details regarding allele frequency, pathogenicity score, and the American College of Medical Genetics and Genomics classification are provided in Supplementary Table 2.

#### 3.1.1 Patient 1: *PLG* mutation K38E

The patient is a 49-year-old woman who has had 5 episodes of TMA treated at an outside hospital, beginning at 32 years of age. All episodes were similar in presentation with nausea, vomiting, and abdominal pain for 1 day, with laboratory data significant for a microangiopathic hemolytic anemia, severe thrombocytopenia, and AKI requiring hemodialysis (HD). During each of these episodes, she required HD for 2 to 4 weeks with subsequent complete renal recovery. During the first 2 events, her diagnosis was presumed to be TTP, and she was treated with plasmapheresis, but an ADAMTS-13 activity level was not obtained. During the third episode, an ADAMTS-13 value was obtained and was found to be normal. Given this, a clinical diagnosis of aHUS was made, and she was started on complement blockade therapy with eculizumab. However, despite being on eculizumab therapy, she developed another episode of recurrent TMA 5 years later. She was then switched to ravulizumab. Genetic testing revealed a *PLG* mutation, and she was then referred to our institution for evaluation. Because of the finding of a *PLG* mutation and lack of response to complement blockade therapy, we started prophylactic anticoagulation with rivaroxaban, given the role of plasminogen in fibrinolytic system, and discontinued ravulizumab. No documented TMA recurrences have occurred since starting anticoagulation therapy almost 2 years ago.

#### 3.1.2 Patient 2: *PLG* mutation R523W

The patient is a 64-year-old woman who came to OSU for a second opinion due to her history of recurrent TMA events. Her first episode occurred almost 9 years prior at the age of 55 years, and since then, she had 4 recurrent TMA episodes. All episodes were similar in presentation with acute onset of nausea and vomiting and dark colored urine with laboratory tests concerning for a microangiopathic hemolytic anemia, severe thrombocytopenia, hematuria, and AKI requiring HD. Her renal function recovered quickly, and she was independent of HD in <1 month during each of these events. She was treated at an outside hospital for each of these episodes, with a presumed diagnosis of TTP vs autoimmune hemolytic anemia. ADAMTS-13 was not checked, and it is unclear why it was never obtained. She never received complement blockade therapy with eculizumab. She was referred to us after the most recent TMA episode, and we obtained genetic testing, which came back positive for a *PLG* mutation. She was started on prophylactic anticoagulation with apixaban in an attempt to prevent TMA recurrence. She has been on anticoagulation for 1 year now, with no recurrence thus far.

#### 3.1.3 Patient 3: *PLG* mutation R261H

The patient is a 43-year-old woman who initially presented with AKI, microangiopathic hemolytic anemia, and a normal platelet count. There was no documented or known trigger for this event. She was started on oral steroids given initial concern for autoimmune hemolytic anemia, and she required HD for AKI. A kidney biopsy was obtained with findings consistent with an acute or chronic TMA. She was then started on eculizumab. A TMA gene panel was obtained and showed *PLG* mutation as well as homozygous deletion of *CFHR1* (heterozygous deletion of *CFHR3*-*CFHR1* on one allele and a heterozygous deletion of *CFHR1*-*CFHR4* on the other allele). Anti-factor H antibody testing was negative (<50 AU). No improvement in renal function was noted even after 6 months on eculizumab. She continues to be HD dependent with no further recurrent TMA episodes. She is not on any anticoagulation therapy at this time.

#### 3.1.4 Patient 4: *PLG* mutation R89K

The patient is a 56-year-old woman who presented with acute onset of abdominal pain and confusion. Her laboratory data were significant for microangiopathic hemolytic anemia, moderate thrombocytopenia, and AKI. She was suspected of having TTP initially, but the ADAMTS-13 level was normal, suggestive of a TMA due to another etiology. Eculizumab therapy was initiated for a possible CM-TMA. A TMA gene panel revealed a *PLG* mutation. Eculizumab was stopped after 1 year due to no improvement in kidney function. She did not require HD, but creatinine remained high at 2.6 mg/dL. There were no documented TMA recurrences since the first episode as of last follow-up in June 2023.

#### 3.1.5 Patient 5: *PLG* mutation T114M

The patient is a 40-year-old woman who had a TMA episode at the age of 36. She presented to an outside hospital with AKI. Laboratory data were significant for microangiopathic hemolytic anemia and AKI with a normal platelet count. Renal biopsy was obtained that demonstrated acute TMA findings. She then underwent extensive evaluation for the etiology of TMA, and additional laboratory tests were positive for lupus anticoagulant and β-2 glycoprotein I IgG antibodies, leading to a presumed diagnosis of antiphospholipid syndrome-associated TMA. Her AKI improved after starting unfractionated heparin during admission, and she was eventually transitioned to warfarin. We repeated the lupus anticoagulant and β-2 glycoprotein IgG and IgM antibodies tests after >12 weeks and all were negative, ruling out antiphospholipid syndrome as the likely diagnosis. A TMA gene panel revealed *PLG* mutation. Consequently, anticoagulation with warfarin was continued. No recurrent TMA episodes have been noted over the past 5 years while she has been on warfarin.

#### 3.1.6 Patient 6: *PLG* mutation P372A

This patient is a now 40-year-old man who had the first episode of TMA at the age of 5 years. He presented with microangiopathic hemolytic anemia, severe thrombocytopenia, and AKI. There was no documented or known trigger for this event, and ADAMTS-13 activity was normal. He had complete hematologic and renal recovery within a few days without complement blockade therapy. TMA genetic analysis showed a pathogenic *MCP* mutation and *PLG* mutation of unknown significance. Over the next 10 years, he has had 5 to 6 self-limited recurrent TMA episodes. His clinical course is well explained by the *MCP* mutation (younger age at TMA onset, multiple self-limited recurrences), but the role of the *PLG* mutation remains unclear. Hence, this patient was not started on anticoagulation therapy.

### 3.2 Plasminogen variant structural mapping results

Most of the *PLG* mutations identified in our cohort were deemed as variants of unknown significance by the reference laboratory report. To better understand the functional consequence of these mutations, we performed structural mapping of these variants on the closed confirmation of the plasminogen molecule, solved by Law et al. [11].

**Figure 2:**
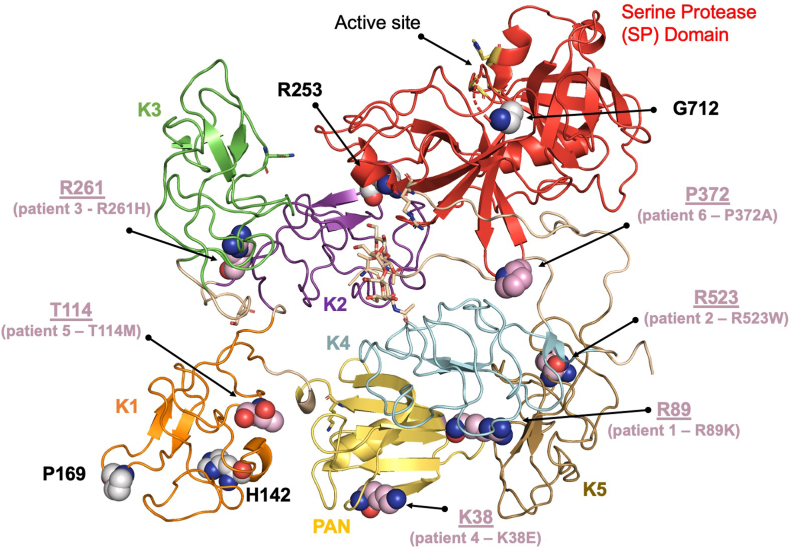
Structural mapping of the plasminogen variants on the closed form of type II Glu-plasminogen.

A total of 10 variants were analyzed based on previously reported *PLG* variants [10,13] (K38E, H142Q, P169S, R253H, R523W, and G712R), and the 4 new ones found in our cohort (R89K, T114M, R261H, and P372A). Variants were mapped on the closed form of type II Glu-plasminogen (Figure 2), which is the resting form of plasminogen circulating in the blood. Mutated residues found in our cohort are shown as pink spheres. Mutated residues already reported in the literature but not found in this study are shown as gray spheres.

The mature circulating form of plasminogen is made up of 791 amino acids, which are organized into 7 domains [14]. These domains include 1 PAN domain, 5 kringle domains (K1, K2, K3, K4, and K5), and the SP domain, which contains the active site. Structural mapping of the variants revealed that the mutations are not clustered in one area but rather are distributed in different parts of the molecule. With the exception of G712, which is located in the SP domain (marked in red), the remaining mutations are located in the auxiliary domains and can be divided into 3 functional groups. Group 1 is composed of residues H142 and P169 in K1. Histidine and proline are unique among amino acids due to their sensitivity to microenvironment (histidine) and the structural constraints (proline). Mutations of these residues are likely to play a role during protein folding, affecting the circulating levels of plasminogen. Group 2 is composed of residue K38 in the PAN domain and residue R523 in K5. These residues are solvent-exposed and do not make apparent contact with nearby residues. However, mutation of these residues might impact the ability of plasminogen to interact with fibrin, cell receptors, or protein cofactors, which stimulate the conversion of plasminogen to plasmin [15]. They could also result in compromised binding of plasmin to macromolecular substrates. Group 3 is composed of residues R89 in the PAN domain, T114 in K1, and R253 and R261 in K2. These residues, unlike residues belonging to group 2, are situated at domain-domain interfaces, stabilizing adjacent domains. These interfaces may be critical to maintaining the closed conformation of plasminogen. Mutation of these residues might, therefore, result in the destabilization of the closed form in favor of alternative structures, potentially affecting the rate at which plasmin is formed. Last, like most other variants, P372 is located outside the protease domain specifically in the linker region connecting K4 and K5. This linker is of particular interest because it is proximal to the protease domain and faces the activation loop. Mutations of this residue may influence loop dynamics and, consequently, the efficiency of plasminogen activation to plasmin. However, given the inherent flexibility of loops, predicting their behavior is very challenging.

When looking at our patient cohort and correlating the phenotypic and genotypic findings, the variants of patients 1 (K38E) and 2 (R532W) belong to group 2, while the variants of patients 3 (R261H), 4 (R89K), and 5 (T114M) belong to group 3. Notably, patients with group 2 variants had a more severe clinical course with more severe thrombocytopenia at presentation and multiple recurrences. Patients with group 3 variants had a lesser degree of thrombocytopenia and no TMA recurrence since the initial event. The clinical course for patient 6 can be well explained by the *MCP* mutation, and role of the *PLG* mutation remains challenging to predict. None of the patients in our cohort carried variants belonging to group 1.

## 4 Discussion

In this study, we describe the clinical features and treatment outcomes of 6 individuals with TMA who were found to have mutations in *PLG*. We found 3 new TMA-associated *PLG* mutations, namely, R89K, T114M, R261H, and P372A. We observed that the patients with *PLG* mutation–associated TMA did not respond effectively to complement blockade therapy. Although the sample size is small, an interesting finding was that patients who were on anticoagulation therapy had TMA resolution and no further recurrent TMA episodes, suggesting there may be a role for anticoagulation in these cases to prevent microthrombi, given the function of plasminogen in the coagulation pathway.

The role of *PLG* variants in the pathogenesis of TMA was first described by Bu et al. [10] in a single-center study of 36 patients with aHUS. In their cohort, 84 variants were identified across multiple genes implicated in aHUS pathogenesis, including several pathogenic *PLG* mutations, supporting a potential role for *PLG* in aHUS. The only overlapping variant between their cohort and ours was c.112 A>G (K38E), observed in our patient 1. However, phenotypic details were not reported for their cohort. Notably, although Bu et al. [10] classified this variant as associated with “traditional” CM-TMA, patient 1 in our study experienced multiple relapses despite long-term complement blockade therapy, suggesting an alternative or additional pathogenic mechanism. Although genes involved in the complement and coagulation pathways were affected in our cohort, recent evidence also suggests that *PLG* variants may co-occur with mutations in noncomplement genes such as *MMACHC*, potentially contributing to TMA via dual or modifying mechanisms [16]. Furthermore, a separate study investigating the role of *PLG* in spontaneous thrombosis identified 4 variants potentially associated with a prothrombotic state, underscoring the role of impaired fibrinolysis and the potential benefit of anticoagulation in patients with *PLG* mutations [12]. Interestingly, 5 of the 6 individuals in our cohort were female. While this may be coincidental due to the small sample size, prior studies have reported sex-based differences in components of the fibrinolytic system such as tissue plasminogen activator and plasminogen activator inhibitor-1, which may be influenced by hormonal regulation [17]. Although direct evidence of sex-specific differences in plasminogen function is limited, our findings raise the possibility that sex-related biological or hormonal factors may contribute to phenotypic variability in *PLG*-associated TMA, warranting further investigation in larger cohorts.

We performed structural mapping of the *PLG* variants to understand the location of these variants and potential mechanism of action. We found that most of the mutations were located in the auxiliary domains and not the SP domain where the active site is found (Figure 2). Moreover, our data suggest that the location of the variant within the plasminogen molecule may determine the clinical course of patients with group 2 variants (residue K38 in the PAN domain and residue R523 in kringle-5) who have a more severe clinical phenotype. As this was a retrospective study, we are unable to comment on the plasminogen antigen and activity levels in these patients. Nonetheless, these findings highlight the crucial role of auxiliary domains in regulating plasminogen function, warranting future investigations to determine the functional impact of these mutations on plasminogen activity. This could make plasminogen activity a useful marker for early identification of these mutations. Given the molecular plasticity of plasminogen, applying recent advances in biophysical and structural biology methodologies for studying coagulation proteins [[18], [19], [20], [21]] will be critical in understanding how the mutations identified in this study impact the shape plasminogen takes in plasma, which in turn affects the conversion of plasminogen to plasmin, as well as the interaction of plasminogen and plasmin with cofactors and macromolecular substrates. Understanding these details could result in plasminogen activity tests for early identification of TMA patients who would benefit from anticoagulation. This basic knowledge is also critical for the development of targeted plasminogen therapeutics.

With growing recognition of the role of complement pathway in TMA pathogenesis, the clinical approach to TMA has evolved significantly over the past decade. A notable finding in our cohort, however, was the lack of response to complement blockade therapy and high rates of recurrence among patients with *PLG* variants. Of the 6 patients in our cohort harboring *PLG* variants, 3 were treated with eculizumab, a complement inhibitor, but demonstrated minimal to no clinical response. Moreover, recurrence rates were unaffected by complement blockade, in contrast to patients with CM-TMA, who rarely experience relapses while on complement blockade therapy [22,23].

Hyvärinen and Jokiranta [24] also investigated the role of plasminogen in TMA pathogenesis using a human endothelial cell model. They found that plasminogen did not interfere with complement-mediated cell lysis or prevent the deposition of activated complement proteins on endothelial surfaces, indicating no regulatory role for plasminogen in the complement cascade. Instead, they observed a significant inhibitory effect of plasminogen on platelet aggregation, suggesting that *PLG* mutations may drive TMA through impaired fibrinolysis and excessive thrombus formation, rather than through complement dysregulation [24]. Based on this data, anticoagulation could be hypothesized to be a potential treatment option via attenuation of the formation of the thrombus given presumed defect in fibrinolysis in patients with *PLG* mutations. In our cohort, 2 patients were started on anticoagulation after identification of a *PLG* variant, with 1 patient already on anticoagulation at the time of genetic diagnosis. While we acknowledge this is a very small sample size and the follow-up period since initiation of anticoagulation is not very long, these data suggest the potential role of anticoagulation for prevention of TMA recurrence in the subset of individuals with TMA and *PLG* variants. Another potential therapeutic approach is plasminogen replacement therapy. In an animal model of TMA caused by cholesterol crystal embolism, Glu-plasminogen therapy was shown to be effective [25]. A human plasma-derived plasminogen concentrate (Ryplazim) has been Food and Drug Administration-approved for the treatment of type 1 plasminogen deficiency; however, it requires frequent dosing every 2 to 4 days [26]. Given the burden of frequent administration, we initially opted for anticoagulation in the 2 patients for whom a treatment decision was necessary. Additional studies are needed to determine the efficacy and practicality of plasminogen concentrate therapy in the treatment of *PLG* mutation-associated TMA.

Consistent with TMA being a rare disorder, our study has several limitations. First, our sample size of individuals with *PLG* variants is small, and these findings are observational in nature and thus need to be validated in a larger study. Second, we were unable to obtain plasminogen antigen or activity levels in patients with *PLG* mutations, which would have helped determine whether a functional plasminogen deficiency was present. While this does not detract from our genetic findings, such data could have provided additional support for the pathogenic role of *PLG* variants in TMA. Third, some patients were initially evaluated and managed at outside institutions and referred to our center for second-opinion consultation, which may have contributed to heterogeneity in initial diagnostic work-up and treatment approaches. Finally, the targeted gene panel used for genetic testing does not include several more recently implicated genes in TMA pathogenesis, such as *TSEN2*, *EXOSC3*, and *MTR* [27].

In summary, this study is noteworthy for implicating *PLG* variants in TMA pathogenesis and describing the clinical phenotype of those with *PLG* variants associated with TMA. Our findings suggest that there may be a role for anticoagulation in this subgroup of TMA, but larger prospective multicenter studies are needed to further confirm and validate our findings. Our data serves as a clinical observation and lays the groundwork for future hypothesis-driven research. Future studies are warranted for identification of early clinical markers of *PLG* mutation–associated TMA, so these patients can be identified during their initial presentation and their treatments targeted appropriately to the pathogenesis of their TMA.
