# Supplementary material for: Plasminogen mutation–associated thrombotic microangiopathy and role of anticoagulation: a single institution case series
Source: Res Pract Thromb Haemost. 2025 Aug 13;9(6):103012. doi: 10.1016/j.rpth.2025.103012 (PMC12570182; doi:10.1016/j.rpth.2025.103012)
Supplement: Supplementary Material [file mmc1.docx]

**Supplemental table 1: Genes included in the TMA genetic testing**

| *CFH* |
| --- |
| *CFI* |
| *CFB* |
| *CFHR5* |
| *C3* |
| *MCP* |
| *THBD* |
| *ADAMTS13* |
| *PLG* |
| *DGKE* |
| *MMACHC* |
| *G6PD* |
| *WT1* |
| *MLPA* |

**Supplemental table 2: Details regarding *PLG* variants with ACMG classification**

| **Patient** | ***PLG* variant** | **ACMG classification** | **In silico pathogenicity score** | **Allele frequency in gnomAD** |
| --- | --- | --- | --- | --- |
| 1 | c.112A>G, p.Lys38Glu | Pathogenic and Likely pathogenic | - | 0.2892% in European (non-Finnish) population |
| 2 | NM_000301:3c.1567C>T, p.Arg523Trp | VUS | Predicted pathogenic by 1 of the 6 available pathogenicity prediction algorithms | 1.229% in European (non-Finnish) population |
| 3 | NM_000301.3:c.782G>A, p.(Arg261His) | VUS | Predicted pathogenic by 5 of the 6 available pathogenicity algorithms (SIFT, GERP++RS, PolyPhen, Mutation Taster, PhyloP). The CADD score is 27.7. | 0.4178% in European (non-Finnish) population |
| 4 | NM_000301.3:c.266G>A, p. Arg89Lys | Likely benign | Predicted pathogenic by 1 of the 6 available pathogenicity prediction algorithms | 1.144% in European (non-Finnish) population |
| 5 | NM_000301:c.341C>T, p.(Thr114Met) | VUS | Predicted pathogenic by 5 of the 6 available pathogenicity algorithms, GERP++RS (conserved, score 4.01), PhyloP (conserved, score 2.519), PolyPhen2HDIV (probably damaging, score 1), SIFT (damaging, score 0.01) and Mutation Taster (disease causing, score 0.996072) | 0.05013% in east Asian population |
| 6 | NM_000301:c.1114C>G,p.Pro372Ala | VUS | Predicted pathogenic by 2 of the 6 available pathogenicity prediction algorithms, GERP++RS (conserved, score 4.52) and PhyloP (conserved, score 2.529000) | Unknown |

Abbreviations used: ACGM- American College of Genetics and Genomics; VUS- variant of unknown significance
